# Robotics in arthroplasty: how good are they?

**DOI:** 10.1016/j.jseint.2024.09.005

**Published:** 2024-09-21

**Authors:** Maxim Vanderstappen, Johannes H.M. van Ochten, Olivier Verborgt

**Affiliations:** a Orthopaedic Center Antwerp (ORTHOCA), AZ Monica, Antwerp, Belgium; b Department of Orthopaedic Surgery and Traumatology, University Hospital of Antwerp, Antwerp, Belgium; c Faculty of Medicine and Health Sciences, Department of Rehabilitation Sciences and Physiotherapy (REVAKI), Research Group MOVANT, University of Antwerp, Antwerp, Belgium

**Keywords:** Shoulder, Arthroplasty, Technology, Innovation, Robotics, Future

## Abstract

Robotic systems have emerged as indispensable allies in the surgical arena, revolutionizing traditional practices and enhancing the capabilities of health-care professionals. Far removed from the autonomous robots depicted in science fiction, these robotic systems operate under the skilled guidance of surgeons, who control specialized instruments via intuitive consoles and often with the help of robotic product specialists. This symbiotic relationship between man and machine has propelled robotic surgery to the forefront of medical innovation, offering a plethora of benefits that transcends the limitations of conventional surgical techniques. In orthopedic surgery, robotic-assisted knee and hip arthroplasty has experienced rapid growth, and the next field of interest is, without a doubt, shoulder arthroplasty. Digitalization and the use of robotics in shoulder arthroplasty has drawn a lot of attention with the goal to improve the correction of joint deformities and component implantation, possibly leading to enhanced patient outcomes. This next evolution in surgical technology aims to make shoulder replacements more accurate and reproducible in both easy and challenging shoulders and usable by both low-volume and highly experienced surgeons. Nevertheless, robotic-assisted shoulder arthroplasty presents significant challenges related to cost, implant selection, training, and long-term efficacy. Addressing these challenges will require collaboration between surgeons, manufacturers, and regulatory bodies to ensure the safe and effective integration of robotic technology into orthopedic shoulder practice.

The modern hospital setting, intertwined with engineering and technology, has undergone a profound transformation, reshaping the landscape of health-care delivery. Every corner of the hospital, from the bustling operating rooms to the meticulously controlled environments of intensive care units and laboratories, bears witness to the pervasive presence of cutting-edge technology. At the forefront of this technological revolution stands robotic-assisted surgery, a groundbreaking approach that seamlessly merges the precision of machines with the expertise of human surgeons, redefining the standards of medical excellence and patient care.

Robots are still relatively new in the medical field. The first direct interventional support by a robotically assisted surgical system on a human patient occurred in 1985: A PUMA-200 industrial robot positioned and locked a biopsy channel during a computed tomography-guided brain biopsy in neurosurgery.^37^ Since then, extensive innovations have been made in robotic surgery, driven by the aspiration of improving the surgeon’s accuracy and precision during more challenging procedures.^46^ In surgery, the Food and Drug Administration approval of the da Vinci robotic system (Intuitive Surgical; Sunnyvale, CA, USA) for laparoscopic procedures was the cornerstone for the widespread application of robotic-assisted surgery that is present to date.^37^ The significant advantages of binocular endoscopic vision, providing a true 3D experience, combined with ergonomic handles that replicate natural hand movements, have driven the widespread adoption of robotic surgery in general surgery, urology, and gynecology.^11^^,^^37^^,^^46^ Reduction of patient morbidity, decrease of hospital stay length, and faster rehabilitation turned robotic-assisted prostatectomy and partial nephrectomy into the standard of care in well-resourced medical centers.^25^^,^^29^^,^^36^^,^^43^^,^^44^

In the field of orthopedic surgery, the incorporation of robotic-assisted technology has announced a new era characterized by precision and efficiency. From hip and knee joint replacements to spinal surgery, robotic systems have transformed the landscape of orthopedic procedures, offering unmatched accuracy and consistency. The journey began with the introduction of ROBODOC in 1992, a robotic assistance device designed for total hip arthroplasty (THA) with the goal of enhancing the fit and positioning of the femoral component. Since then, the use of robot-assisted surgery for hip, and especially knee arthroplasty has rapidly expanded in recent years, with most marketing-leading companies now offering a robotic device.^21^^,^^31^ There are several incentives for this rapid growth in utilization. First, there remains a great margin for improvement in clinical patient outcomes and satisfaction after total knee arthroplasty (TKA).^31^ Despite advancements in implant design and surgical techniques, around 20% of patients remain unsatisfied after 1 year postoperatively.^2^^,^^21^ One of the main cited goals of robotic assistance is to reduce this patient dissatisfaction rate by improving procedure accuracy and precision. The former means that the achieved component position is near the desired target, and the latter means that the component position can be achieved in a reproducible fashion.^52^ As studies have shown that conventional jig-based instrumentation is frequently associated with component malalignment in both the coronal and sagittal plane, improvements in this area may be achievable with robotic-assisted TKA (raTKA) and robotic-assisted unicompartmental knee arthroplasty (raUKA).^18^ Furthermore, traditional intraoperative gap balancing and ligament tensioning remain subjective.^22^ Depending on the system, certain robots aid in intraoperatively evaluating the limb alignment and joint stability, protecting soft tissues by utilizing boundary control, and allowing for case-by-case data collection.

Due to high patient satisfaction and excellent survival of modern hip implants, the margin for improvement by utilizing robot-assisted total hip arthroplasty (raTHA) may be less appreciable.^13^^,^^34^ Nevertheless, the prevalence of raTHA increased from less than 0.1% in 2008–2.1% in 2018.

## Benefits of robotics in arthroplasty

**Figure 1:**
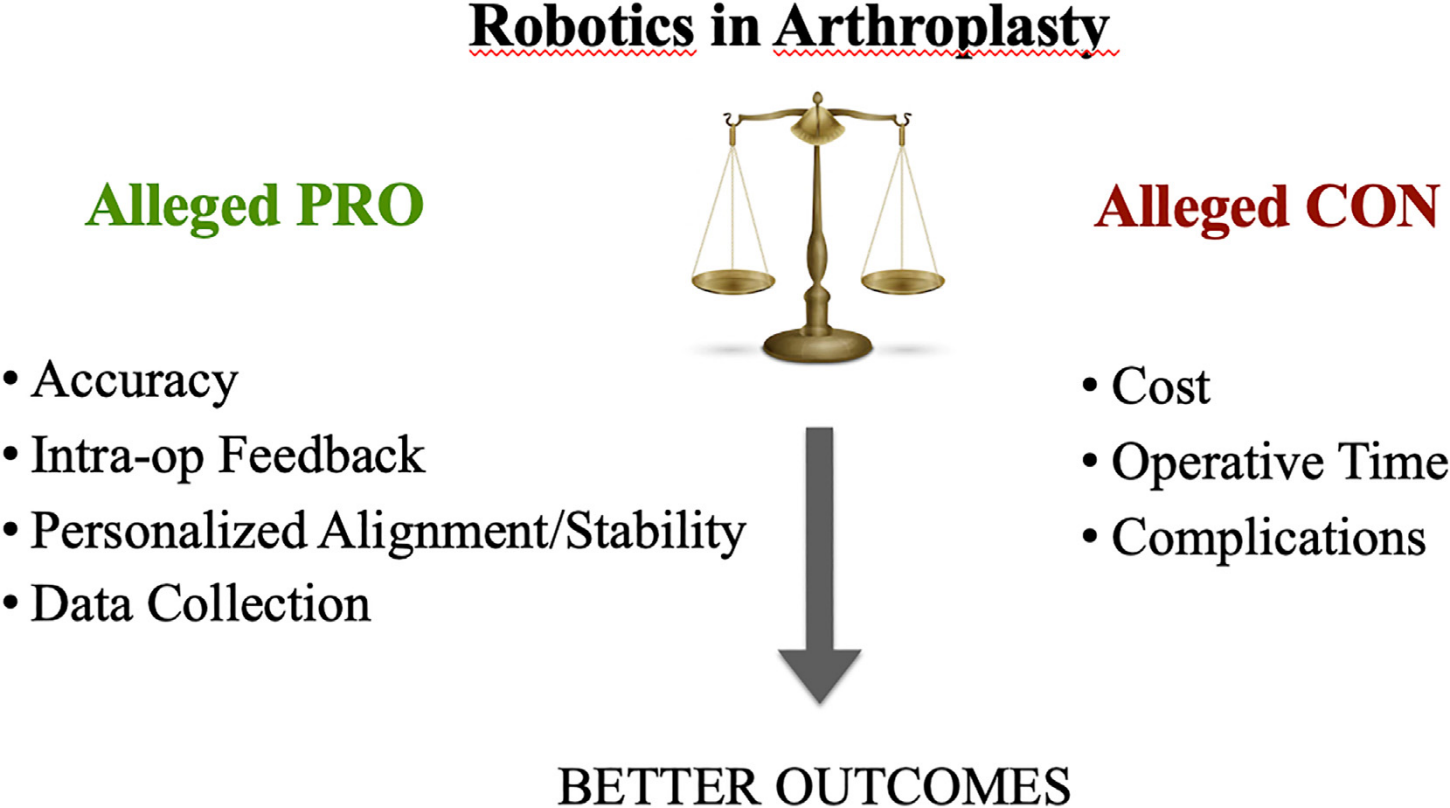
Pros and cons of robotics in arthroplasty.

Despite all discussions and weighing of pros and cons, orthopedic robots have experienced a substantial growth from a business perspective, with nearly 3000 robots now active in operating rooms worldwide. However, the crucial question lingers: how effective are they? Delving into the existing literature on raTKA and THA, it’s important to acknowledge that much of the current research is conducted by key opinion leaders in the field, introducing potential bias.^9^^,^^39^ Alleged advantages include enhanced accuracy, real-time feedback during surgery, personalized alignment and stability, and comprehensive data collection. Conversely, perceived drawbacks encompass expenses, prolonged operative time, and potential complications, leading to uncertainty regarding whether these technologies genuinely yield superior outcomes (Fig. 1).

### Accuracy and precision

Most current literature comes from TKA where, in summary, raTKA has demonstrated improvements in accuracy and reproducibility in component alignment, reducing outliers compared to manual techniques.^4^^,^^16^^,^^35^^,^^40^^,^^55^ Studies by Bellemans et al showed accuracy within 1° to a neutral alignment in the coronal and sagittal planes across 25 cases. Cadaver studies utilizing robotic arm–assisted systems by Hampp et al exhibited improved accuracy and precision in bone cuts and implant placement, while Seidensein et al reported more precise and reproducible bony resections. A recent meta-analysis reported that raTKA resulted in reduced differences in preoperative and postoperative posterior condylar offset, increased accuracy in femoral and tibial component positioning, and a narrower mean error range in placement. Similar radiographic outcomes were observed for raUKA.

Early reported benefits include less pain, less time to straight leg raise, shorter length of stay, and improved WOMAC scores, attributed to better preservation of the soft tissue envelope, reduced releases, and diminished local inflammatory responses with raTKA systems.

The superiority in accuracy and precision does not directly translate to improvements in clinical outcomes in medium to long-term follow-up compared to conventional jig-based TKA.^6^^,^^21^^,^^26^^,^^41^^,^^42^^,^^55^ Most clinical trials and systematic reviews fail to report clinically significant differences between both techniques beyond the initial postoperative period.^27^ Unicompartmental knee arthroplasty, however, may benefit more during extended follow-up from the increase in surgical accuracy as Batailler et al demonstrated an increase in implant survival (95% in raUKA vs. 91% in conventional unicompartmental knee arthroplasty) and reported that 86% of the revisions in the conventional group were attributed to implant malposition or limb malalignment compared to 0% in raUKA.^3^^,^^42^

Similar to the improved radiographic alignment in robotic knee arthroplasty, several studies report significantly improved acetabular component orientation compared to regular THA.^24^^,^^42^ Kamath et al concluded that cup inclination and the percentage of cups placed within the Lewinnek and Callanan safe zones improved significantly while a lower risk of outliers was present.^20^ Domb et al found that 100% and 92% of acetabular components (vs. 80% and 62% in conventional THA) were placed within the Lewinnek and Callanan safe zones, respectively.^12^ Furthermore, as measured on postoperative computed tomography scans, in raTHA femoral length and offset showed low deviation from the planned values, and significant correlations were found between preoperative and postoperative acetabular inclination, version and femoral anteversion.^32^ Most clinical outcome studies, however, show equivalent results between raTHA and conventional techniques, and high-level evidence on modern robotic hip systems is scarce.^23^^,^^45^

### Learning curve and complications

As with most new techniques, there will be a learning curve associated with robotic shoulder arthroplasty.^54^ The requirement for extensive training and the learning curve associated with robotic-assisted procedures can lead to prolonged operating times during the initial phases of implementation. This not only increases surgical expenditures but also necessitates additional resources for training the surgical team. However, it is anticipated that over time, as surgeons become proficient with the technology, the time investment will become more balanced. Moreover, data on rTKA suggest that the implementation of robotic assistance becomes time neutral over time.^1^^,^^28^

Literature highlights the significance of pin tracker complications in raTKA, raising concerns about their impact on surgical accuracy and effectiveness. These perioperative challenges encompass pin migration or breakage, which disrupt the tracking system, compromising the precision of bone cuts and implant positioning. Pin breakage may necessitate intraoperative adjustments or even abandoning the robotic-assisted procedure, prolonging surgical duration and potentially causing discomfort for patients. Additionally, postoperatively, soft tissue irritation at pin sites can induce pain and delay wound healing, elevating the risk of infections and other complications. Although rare, bone weakening due to pin placement may cause fractures. It is crucial for surgeons to be aware of these complications and address them diligently to ensure optimal outcomes in raTKA. Overall, robotic-assisted surgeries typically do not present more complications when compared to conventional procedures.^5^^,^^10^^,^^21^^,^^33^^,^^47^

### Cost/benefit

Robotic technology is associated with high additional costs. These may be attributed directly to the installation and the maintenance costs of the device itself or indirectly due to disposables, software updates, service contracts, and necessary preoperative imaging. Furthermore, prolonged operating times during the learning phase and training of the surgical team can result in increased expenditures. These costs may be partially offset if robotic surgery proves to reduce opiate analgesia consumption, shorten hospital stay and reduce readmission rates, and reduce discharges to postacute care rehabilitation centers compared to conventional arthroplasty.^7^^,^^19^^,^^53^ The benefit, however, always needs to be proven and long-term results are needed to prove lower revision rates.

## Robotic-assisted shoulder arthroplasty

**Figure 2:**
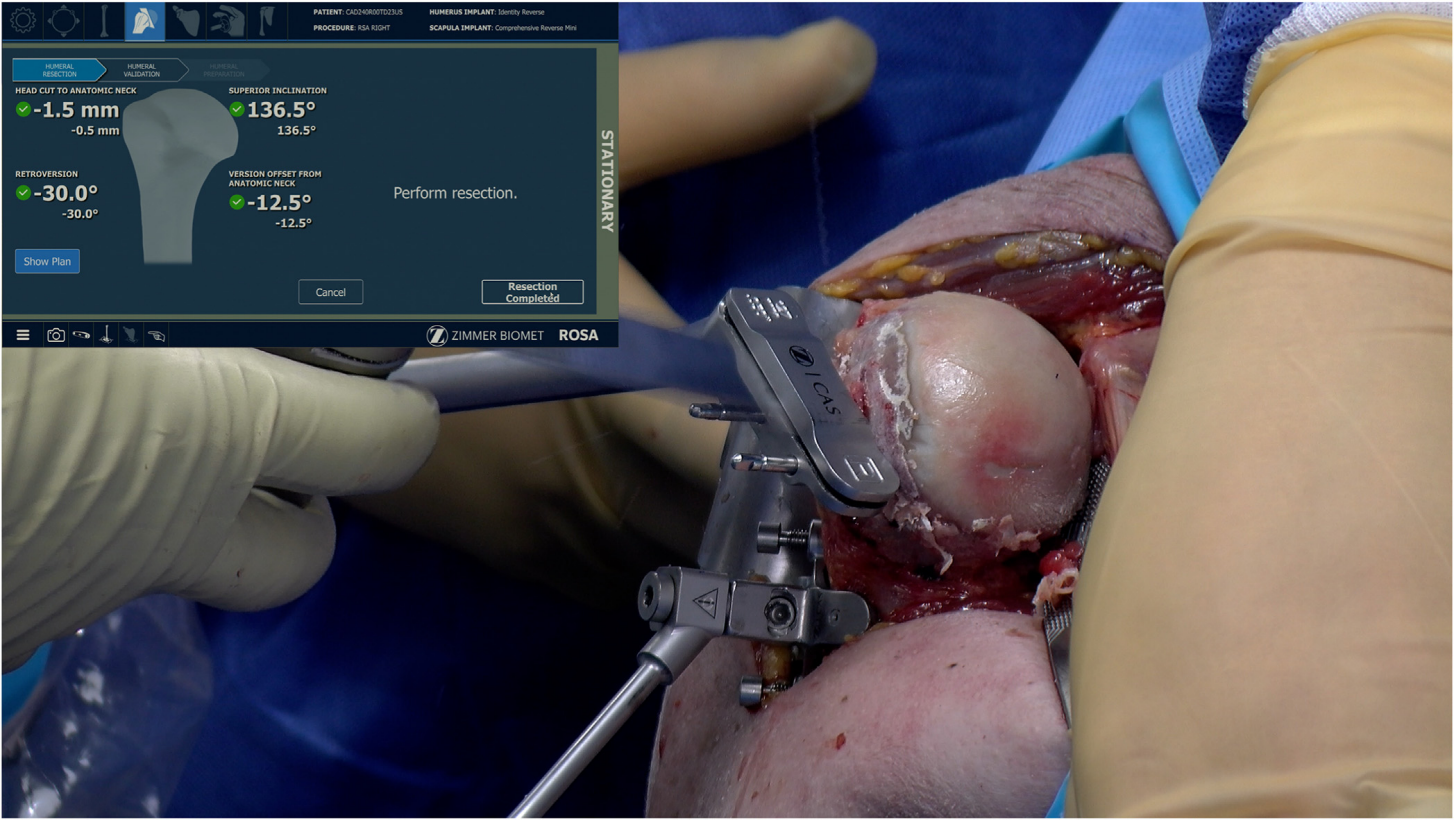
Humeral osteotomy in robotic-assisted shoulder arthroplasty.

**Figure 3:**
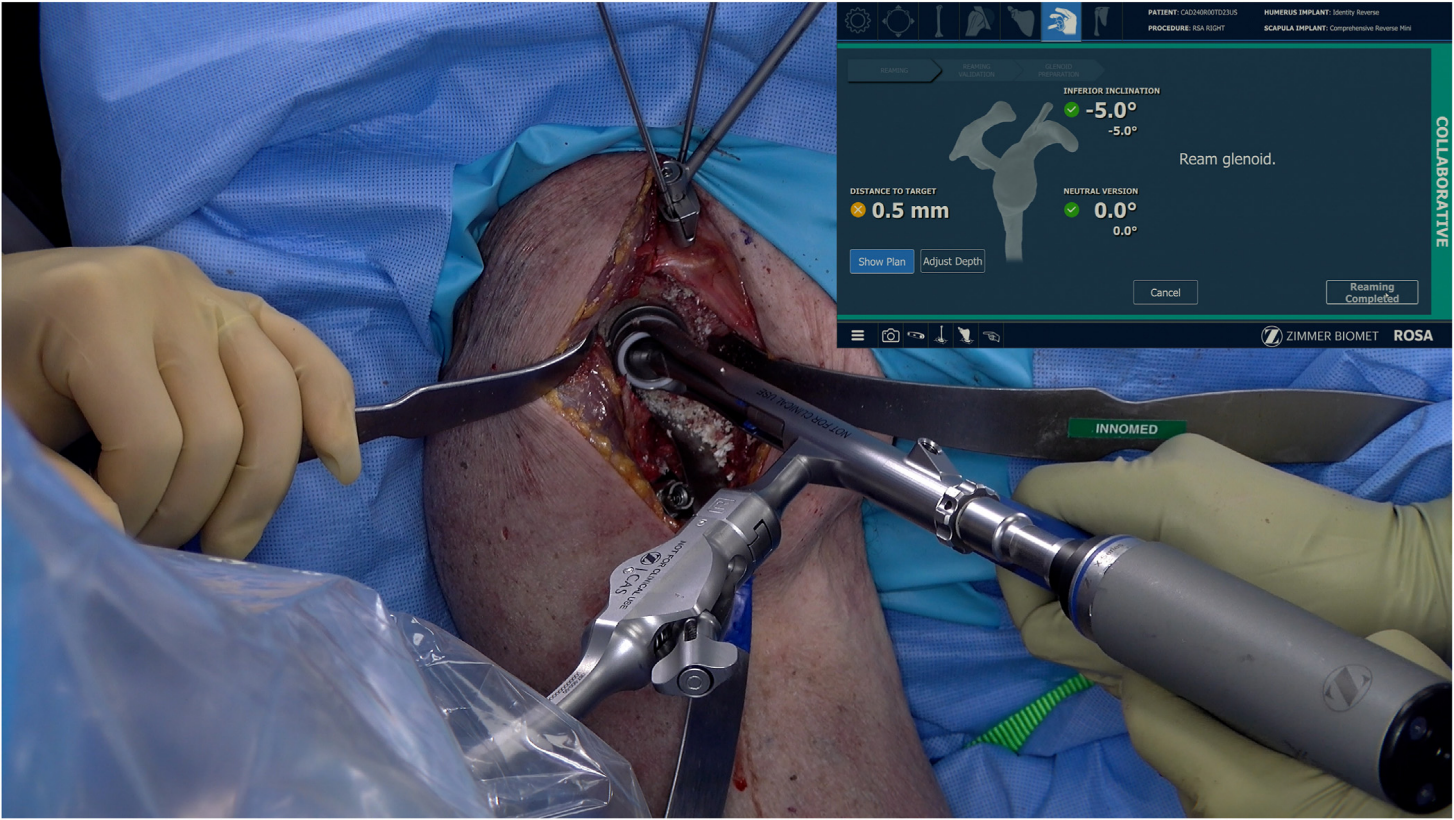
Glenoid reaming in robotic-assisted shoulder arthroplasty.

Shoulder surgeons are currently immersed in new technologies to help plan and execute their shoulder arthroplasties.^14^^,^^15^^,^^38^^,^^48^, ^49^, ^50^, ^51^ Robotic systems could represent the final advancement in the digitalization of shoulder arthroplasty, executing and collecting data throughout the entire process after precise preoperative planning by the orthopedic surgeon. By allowing surgeons to dynamically use available planning software and adapt *ad hoc* specific steps during the surgical procedure itself, the execution of the joint correction and prosthesis implantation can be meticulously fine-tuned. Intraoperative real-time navigation allows the surgeon to plan and execute a patient-specific solution in every case accurately.^48^ It will pave the way for more minimally invasive and soft tissue–sparing techniques without compromising on humeral or glenoid exposure and preparation access. The robot arm with boundary control may assist and restrain the surgeon during the humeral cut and the glenoid reaming process (Figs. 2 and 3). Intraoperative feedback of soft tissue tension would aid in acquiring optimal joint stability. Furthermore, gaining procedural accuracy and reproducibility may be particularly important in shoulder arthroplasty, as previous studies have shown that only 3% of shoulder arthroplasty surgeons performed > 10 procedures per year. Lastly, research may be facilitated and encouraged by supporting intraoperative case-by-case data collection. Findings can be matched and correlated to preoperative and postoperative results. The extensive data acquisition may lead to new guidelines and decision trees for shoulder surgeons on the use of specific techniques and prosthetic implants.

In addition to cost-related challenges, there are practical considerations regarding the use of robotic technology in shoulder arthroplasty. For instance, the placement of glenoid and humerus registration trackers may require additional exposure of the coracoid and proximal humerus, potentially increasing surgical complexity and operative time. Furthermore, the presence of robotic devices, computer screens, and infrared sensors in the operating room may reduce the available working space and lead to instrument crowding. Surgeons and their teams must adapt to these spatial constraints to ensure smooth and efficient surgical workflows. It is also essential to recognize that while robotic assistance can enhance procedural precision, it cannot replace the expertise and judgment of the surgeon. The planning and execution of shoulder replacement surgery still rely heavily on the surgeon’s experience and intuition. While technology can aid in preoperative planning and intraoperative correction of joint deformities, the surgeon’s role in decision-making and final component implantation remains paramount.^48^

## Future directions and opportunities

Looking ahead, the future of robotic-assisted orthopedic surgery is marked by cautious optimism for further innovation and advancement. As technology continues its gradual evolution, robotic systems tailored for orthopedic procedures will undergo refinement, potentially offering a range of enhanced capabilities and functionalities specifically adapted to the difficulties of musculoskeletal surgery. Artificial intelligence and machine learning algorithms may play a role in optimizing surgical workflows, providing real-time decision support, and facilitating personalized treatment planning.^17^^,^^30^

One of the most exciting prospects lies in the potential for harnessing vast amounts of data to create comprehensive databases that serve as invaluable resources for future studies and research in orthopedic surgery. By aggregating data from robotic-assisted procedures, these databases could provide unprecedented insights into surgical outcomes, patient demographics, implant performance, and postoperative complications. Such information could fuel a wealth of future studies, articles, and publications, driving continuous improvement in orthopedic care and surgical techniques. Moreover, the integration of augmented reality and virtual reality technologies holds immense promise for revolutionizing orthopedic surgical education and training.^8^ Imagine a future where orthopedic surgeons can visualize every aspect of implant balancing and surgical technique on a screen, seamlessly integrated with preoperative planning tools. Through immersive augmented reality and virtual reality simulations, trainees can gain hands-on experience in a realistic virtual environment, honing their skills and refining their techniques under expert guidance. This innovative approach to surgical education has the potential to significantly enhance the training experience, accelerate learning curves, and foster collaboration among orthopedic surgeons worldwide.

As the field of robotic-assisted orthopedic surgery continues to evolve, interdisciplinary collaboration will be essential in driving innovation and unlocking new possibilities. Orthopedic surgeons, engineers, data scientists, and health-care stakeholders must work together synergistically to leverage emerging technologies and translate cutting-edge research into concrete clinical applications. By embracing a culture of innovation and collaboration, the future of robotic-assisted orthopedic surgery holds the promise of revolutionizing patient care, advancing surgical practice, and shaping the future of musculoskeletal medicine.

## Conclusions

Orthopedic surgeons are immersed in new technologies to help plan and execute their arthroplasties. For shoulder surgeons, robotic systems may represent the ultimate next digitalization step by mastering the flow process of planning, execution, and case-by-case data collection. Robotic assistance may improve the accuracy and precision of correction and component positioning, soft-tissue balance, joint stability, and research possibilities. Addressing the challenges of cost, training, and long-term efficacy will require collaboration between surgeons, manufacturers, and regulatory bodies to ensure the safe and effective integration of robotic technology into orthopedic shoulder practice.

## Disclaimers:

Funding: No funding was disclosed by the authors.

Conflicts of interest: Olivier Verborgt is a paid consultant and receives royalties from Zimmer-Biomet and serves as executive committee board member of European Society for Surgery of the Shoulder and Elbow S(ECEC/ESSE). All the other authors, their immediate families, and any research foundations with which they are affiliated have not received any financial payments or other benefits from any commercial entity related to the subject of this article.
